# Avian diversity and spatial-temporal distribution pattern of dominant species in Baqing County, Tibet

**DOI:** 10.3897/BDJ.13.e163095

**Published:** 2025-07-23

**Authors:** Jielei Xie, Zhiming Cao, Dandan Wang, Yuqin Liu, Fuxing Huang, Yuanzhen Cui, Xuntao Zhu, Xiaolong Hu, Yan Wang, Yongtao Xu

**Affiliations:** 1 Jiangxi Provincial Key Laboratory of Conservation Biology, Jiangxi Agricultural University, Nanchang, China Jiangxi Provincial Key Laboratory of Conservation Biology, Jiangxi Agricultural University Nanchang China; 2 College of Animal Science and Technology, Jiangxi Agricultural University, Nanchang, China College of Animal Science and Technology, Jiangxi Agricultural University Nanchang China

**Keywords:** bird resources, species diversity, dominant species, relative abundance index, spatial-temporal distribution

## Abstract

The Qinghai-Tibet Plateau, called the “Third Pole of the Earth”, is a typical region with rich avian resources due to its unique geographical environment and climatic characteristics. From August 2023 to May 2024, avian diversity was monitored with infrared camera trapping and transect surveys in Baqing County of the Three-River-Source National Park. A total of 81 bird species in 14 orders and 32 families were surveyed. Four and twelve species were respectively recorded as Class I and Class II nationally key protected animals of China. Based on the relative abundance index (RAI), the most dominant species was *Phoenicurusochruros* (RAI = 2.226), followed by *Prunellarubeculoides*, *Pyrgilaudaruficollis*, *Perdixhodgsoniae* and *Pseudopodoceshumilis*, which all exhibited unimodal diurnal activity peaks. Behaviour rhythm analysis showed daily activity peaks for *Pseudopodoceshumilis* (10:00–15:00 h) and *Phoenicurusochruros* (11:00–16:00 h). Other species with narrower peaks included *Perdixhodgsoniae* (12:00–14:00 h), *Prunellarubeculoides* (13:00–14:00 h) and *Pyrgilaudaruficollis* (11:00–12:00 h). The bird life of Baqing County is primarily dominated by highland elements, Holarctic elements, Palaearctic elements and Himalayan-Hengduan Mountains elements. The highest species richness occurs in habitats exhibiting the species-area effect and edge effect, particularly in grass-shrub ecotones and grass-rock mosaic habitats. This study provides essential baseline data to support avian conservation strategies in the Sanjiangyuan ecosystem.

## Introduction

Three-River-Source National Park (TRSNP), located in the hinterland of the Qinghai-Tibet Plateau, is the vital headwater region for major rivers including the Yangtze, Yellow and Lancang (Mekong) Rivers (Ma et al. 2023). With an average elevation exceeding 4,000 m above sea level, it is acclaimed as the "Water Tower of China" and the "Earth's Third Pole Ecological Barrier" (Yin et al. 2023). As China's first pilot national park system (2013) and one of its first formally established national parks (2021) (National Forestry and Grassland Administration and National Park Administration 2024), its core objective is to protect the integrity and biodiversity of the alpine ecosystem (Wei et al. 2020, Ma et al. 2023). As a biodiversity hotspot on the Qinghai-Tibet Plateau, the region boasts a species C-F index of 0.77 and a G-F index of 0.825 (Cai et al. 2019, Gao et al. 2019), indicating that its biodiversity ranks amongst the highest levels globally for alpine ecosystems. Wildlife populations are not only key components of ecosystem structure and function, but also serve as indicator species reflecting ecosystem health (Gao et al. 2019).

Birds are vital components of ecosystems, playing a crucial role in maintaining biodiversity and ecological balance (Whelan et al. 2015, Gaston et al. 2018). Most bird species possess strong flight capabilities, enabling them to perceive changes in their surroundings and respond promptly. The composition of bird communities in a given area serves as a significant indicator for monitoring environmental health and ecosystem integrity (Cunningham and Johnson 2006). Environmental factors such as altitude, temperature, precipitation, topography, solar radiation and the intensity of human activity can influence the formation and distribution patterns of bird communities to varying degrees (García-Navas et al. 2021). Furthermore, due to resource limitations, sympatric species often partition resources along dimensions such as diet, temporal activity and habitat use to achieve co-existence (Liu et al. 2017, Petalas et al. 2021). Therefore, quantifying the characteristics of species diversity, including its magnitude and spatial distribution, has become a fundamental aspect of research in avian conservation and ecology.

Baqing County, located in the Tangbei Region of TRSNP and administratively under the Tibet Autonomous Region, has an average elevation exceeding 4,500 m. As part of the ecologically fragile zone on the Tibetan Plateau, it is characterised by extreme cold, oxygen deprivation and alpine conditions (Jia et al. 2005). The county's pronounced vertical elevation gradient (over 1,500 m) has created diverse habitats, including alpine meadows, shrublands and wetlands, contributing to high levels of biodiversity (Altamirano et al. 2020). However, climate change has caused an average temperature increase of 0.36℃/decade (Yao et al. 2016). Research indicates that, due to changing precipitation patterns, the habitat of the Black-necked Crane (*Grusnigricollis*) in the Shaluli Mountains has expanded towards higher altitudes (Li et al. 2022). Similarly, the Chinese Grouse (*Tetrastessewerzowi*) is projected to migrate northwards and to higher elevations (Lyu and Sun 2014). Currently, Baqing County faces compounded threats from 11 types of natural disasters, including pika infestations, grassland degradation and snow disasters (Yin et al. 2023). The conflict between human activities and ecological conservation is intensifying, leading to accelerated habitat fragmentation and heightened species loss. While regional checklists exist, fine-scale data on species distribution, habitat use and seasonal abundance within the Tangbei area of TRSNP are critically lacking. This absence of data hinders targeted conservation efforts and effective management planning.

In this study, we used transect survey and infrared camera monitoring to assess the composition and distribution of bird diversity in the Tangbei area (Baqing County) of the TRSNP. We analysed the spatial and temporal distribution characteristics of dominant species of birds. The results can provide a scientific basis for the accurate evaluation of the region's biodiversity, the status of endangered species and the protection and management of the region.

## Methods

### Study area

The project implementation area is located in Baqing County, a critical zone of the TRSNP region in Nagqu Prefecture, Tibet Autonomous Region. Situated in the southern Qiangtang Great Lake Basin along the upper Nujiang River within the eastern Nagqu Plateau of northern Tibet, this area features a north-to-south descending topography with an average elevation exceeding 4,500 m. Its highest point is Mount Bashan Peak at 6,860 m above sea level. The survey focuses on Baqing County's territory within the TRSNP, encompassing the townships of Gangqê, Maru, Jiangmian, Gongri etc. (Ma et al. 2023). Its subarctic semi-humid plateau monsoon climate fosters a composite ecosystem of alpine meadows and mountain valleys. With approximately 2,402 annual sunshine hours (Man et al. 2019), it sustains diverse habitats, including alpine peaks, glaciers, wetlands, grasslands and meadows (Xu et al. 2009), with vegetation showing distinct vertical zonation from forests to meadows and scrublands at higher altitudes under cold climatic conditions (Tan et al. 2020).

Fig. 1

### Avian survey

Two standardised methods were implemented for biodiversity monitoring in Baqing County: (1) The survey transects were mainly set up, based on existing main roads and grassland paths within the survey area, totalling 21 transects with a length of approximately 2000 km of systematic surveys across 4×4 km grid units using ArcGIS-mapped routes, based on terrain/vegetation features. The survey was conducted by combining driving and walking, with driving surveys being the primary method. Along the route, birds encountered on both sides were observed and recorded. Sampling points were set at appropriate intervals for manual inspection to record individual bird counts and habitat types, aiming to obtain more comprehensive and detailed biodiversity data; (2) The deployment of 223 passive infrared radiation (PIR) devices (Yi'an L710) in sample plots established by transect surveys constituted the implementation of infrared camera trapping. These devices were deployed at 100-200 m intervals in each grid. Camera placement followed standards of habitat suitability and animal activity traces (species distribution patterns, wildlife activity frequency and our accessibility), prioritising open landscapes: grassland rock clusters, meadow gullies, summit bare rocks, shrub-grass ecotones and mountain-adjacent flats. Cameras were mounted 0.2–1.2 m (Meek et al. 2016, Yang et al. 2022) above ground on natural structures (rocks, fences, shrubs), with specific height adjustments made within coniferous forests. Operational parameters: the infrared cameras capture photos with pixel counts exceeding 8 million, 3 stills + 30 s video per trigger (Hu et al. 2018), 3-4 months of continuous monitoring per station and synchronised GPS logging. This grid-based system was designed to achieve comprehensive biodiversity surveillance coverage.

### Species identification and statistics

Species in the photographic data were identified and their taxonomic classification was determined primarily using Guide to the Birds of China (MacKinnon 2022) and A Checklist on the Classification and Distribution of the Birds of China (4^th^ Edition) (Zheng 2023). The conservation status of each species was assessed by consulting several key sources: the Red List of China’s Vertebrates (Jiang et al. 2016, Ministry of Ecology and Environment of the People's Republic of China 2023); Convention on International Trade in Endangered Species of Wild Fauna and Flora Appendices I, II and III (CITES Appendices I-III) (CITES 1978); IUCN Red List of Threatened Species (The International Union for Conservation of Nature 2022); and List of Key Protected Wild Animals in China (National Forestry and Grassland Administration and Ministry of Agriculture and Rural Affairs of the People's Republic of China 2021); Biogeographical classifications followed in Zoogeography of China (Zhang 2011). Photographs lacking bird, mammalian or human subjects were excluded during data processing. In the infrared camera monitoring, the camera's capture mode was set to 3 photos plus a 30-second video. Therefore, when the images were unclear, identification could still be confirmed using the video. If both the images and the video show birds without distinct features, they are directly marked as unidentified species (Ridout and Linkie 2009, Viviano et al. 2021, Zanni et al. 2021).

### Avian richness

The dominant species within the avian community were identified by the relative abundance index (RAI), which was the infrared camera capture rate, calculated by determining the number of independent events per species per unit of time (Chen et al. 2019).

\begin{varwidth}{50in}\begin{equation*}
            \mathrm{RAI} = \frac{N}{T} \times 100
        \end{equation*}\end{varwidth}（1）,

where N is the number of species-independent valid photographs taken and T is the total number of valid camera workdays, with valid camera workdays defined as the number of dates between the start of camera placement and the last fieldwork photograph for each camera (Carbone et al. 2006, Liu et al. 2013).

### Spatio-temporal distribution analysis

This study employs the Kernel Density Estimation (KDE) method (Ridout and Linkie 2009), utilising the compareCkern function from the *activity* package (Rowcliffe and Rowcliffe 2016) for data processing and computation: The daily activity rhythms and niche overlap of animals, with a 24-hour data type cycle, convert the original time data of independent valid photos (HH:MM:SS) into decimals (value range 0-1), then into radians to facilitate the analysis of species differences using the kernel density estimation method (Nouvellet et al. 2012). Therefore, the densityPlot() function from the *overlap* package generates the kernel density curves for individual species and the smoothness of the entire curve is adjusted using the adjust parameter (Ridout and Linkie 2009).

To compare niche overlap amongst co-distributed avian species, this study employs the overlapEst() function to calculate the overlap coefficient (Coefficient of Overlapping, Δ) for different species under diurnal activity rhythms (Meredith et al. 2013). The overlap coefficient ranges from [0, 1], where values range from 0, indicating complete differentiation, to 1, indicating complete overlap (Mulekar and Mishra 2000, Schmid and Schmidt 2006).

Altitude, habitat type and geographical location were used to analyse the spatial distribution of birds. Grassland, shrub, rock and coniferous forest were used to define the habitat types or mixed habitat categories. The geographical location was primarily delineated using towns and townships as units.

## Results

### Avian diversity

**Figure 2.**
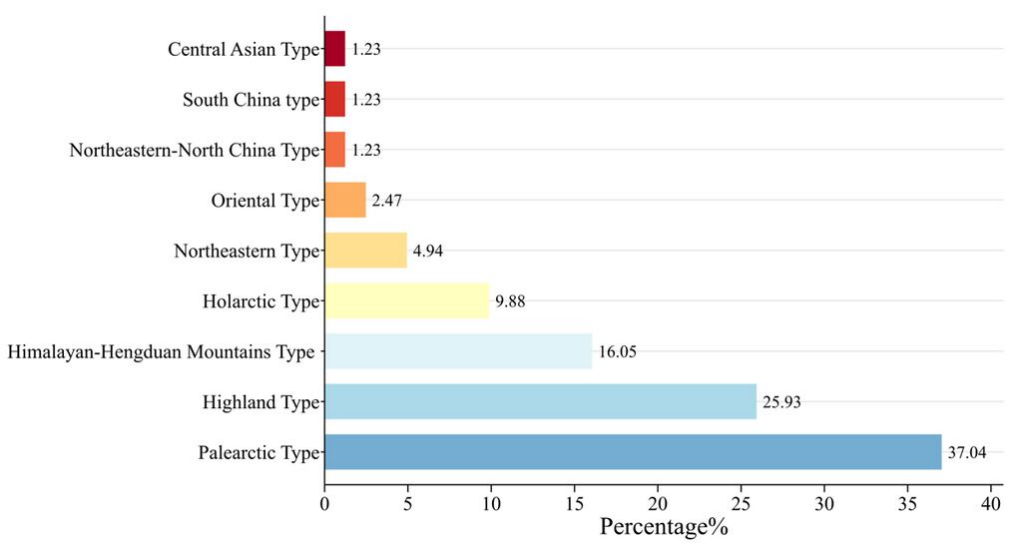
The bird distribution types recorded using the transect survey.

A total of 81 bird species belonging to 14 orders and 32 families were recorded in this survey. Specifically, transect surveys detected 70 species across 14 orders and 31 families, while infrared camera monitoring documented 54 species from seven orders and 22 families. Amongst these, 43 species were recorded by both methods, with 28 species exclusively observed through transect surveys and 11 species uniquely captured by infrared cameras (Suppl. materials 1, 4). Passerines dominated (55 species, 67.90%), contrasted by 26 non-passerine species (32.10%). Fringillidae was the richest family (8 species), followed by Muscicapidae (7), Passeridae (6) and Motacillidae (5), with 27 families containing ≤ 4 species. Distributionally, Palearctic-type birds predominated (30 species, 37.04%), succeeded by Highland-type (21, 25.93%), Himalayan-Hengduan Mountains type (13, 16.05%), Holarctic-type (8, 9.88%), Northeast China-type (4, 4.94%) and Oriental-type (2, 2.47%), while Northeast-North China type, Central Asian type and South China type each had minimal representation (1 species, 1.23%); see Fig. 2 and Suppl. material 1.

### Concerned species

Amongst 81 recorded bird species, four are Class I nationally protected animals in China (under the Wildlife Protection Law) (National Forestry and Grassland Administration and Ministry of Agriculture and Rural Affairs of the People's Republic of China 2021): Black-necked Crane (*Grusnigricollis*), Golden Eagle (*Aquilachrysaetos*), Bearded Vulture (*Gypaetusbarbatus*) and Saker Falcon (*Falcocherrug*). Eleven species are Class II nationally protected animals: Tibetan Eared Pheasant (*Crossoptiloncrossoptilon*), White Eared Pheasant (*Tetraogallustibetanus*), Ibisbill (*Ibidorhynchastruthersii*), Little Owl (*Athenenoctua*), Eurasian Eagle-Owl (*Bubobubo*), Himalayan Vulture (*Gypshimalayensis*), Upland Buzzard (*Buteohemilasius*), Common Kestrel (*Falcotinnunculus*), Tibetan Babax (*Pterorhinuskoslowi*) and White-browed Tit (*Poecilesuperciliosus*).

According to the IUCN Red List of Threatened Species (The International Union for Conservation of Nature 2022), one species assessed as Endangered (EN) is the Saker Falcon (*Falcocherrug*). Black-necked Crane (*Grusnigricollis*), Bearded Vulture (*Gypaetusbarbatus*), Himalayan Vulture (*Gypshimalayensis*), White Eared Pheasant (*Crossoptiloncrossoptilon*), Chinese Babax *(Pterorhinuslanceolatus)* and Tibetan Babax *(Pterorhinuskoslowi*) are classified as Near Threatened (NT). All remaining species are classified as Least Concern (LC).

The Red List of China’s Vertebrates (Jiang et al. 2016, Ministry of Ecology and Environment of the People's Republic of China 2023) identifies one species as Endangered (EN): Saker Falcon (*Falcocherrug*). Three species of birds are vulnerable: Black-necked Crane *(Grusnigricollis*), Golden Eagle (*Aquilachrysaetos*) and Upland Buzzard *(Buteohemilasius)*. Eleven species are Near Threatened (NT): Eurasian Eagle-Owl (*Bubobubo*), Bearded Vulture (*Gypaetusbarbatus*), Himalayan Vulture (*Gypshimalayensis*), Tibetan Snowfinch (*Montifringillahenrici*), White-browed Tit (*Poecilesuperciliosus)*, Streaked Rose (*Carpodacusrubicilloides*), Tibetan Babax (*Pterorhinuskoslowi*), White Eared Pheasant (*Crossoptiloncrossoptilon*), Chinese Babax (*Pterorhinuslanceolatus*), Tibetan Eared Pheasant (*Tetraogallustibetanus*) and Ibisbill (*Ibidorhynchastruthersii)*. The remaining species are classified as Least Concern (LC).

Regarding *CITES* Appendices (CITES 1978), 13 monitored species are listed: Black-necked Crane (*Grusnigricollis*), White Eared Pheasant (*Crossoptiloncrossoptilon)* and Tibetan Eared Pheasant (*Tetraogallustibetanus*) are listed in Appendix I; Little Owl (*Athenenoctua*), Eurasian Eagle-Owl (*Bubobubo*), Bearded Vulture (*Gypaetusbarbatus*), Himalayan Vulture (*Gypshimalayensis*), Golden Eagle *(Aquilachrysaetos*), Upland Buzzard (*Buteohemilasius*), Common Kestrel (*Falcotinnunculus)* and Saker Falcon (*Falcocherrug*) are in Appendix II. There are two species in Appendix III: Common Rosefinch (*Carpodacuserythrinus*) and Ruddy Shelduck (*Tadornaferruginea*) (Suppl. material 1).

### Daily Activity Patterns

Based on the RAI, the dominant avian species within the surveyed area of Baqing County were identified as *Phoenicurusochruros* (RAI = 2.226), *Prunellarubeculoides* (RAI = 1.707), *Pyrgilaudaruficollis* (RAI = 1.427), *Perdixhodgsoniae* (RAI = 1.382) and *Pseudopodoceshumilis* (RAI = 1.364). *Phoenicurusochruros* exhibited the most extensive distribution range, followed by *Prunellarubeculoides*. Owing to the strong flight capability of birds, significant spatial overlap was observed in their distribution patterns, with only limited areas exclusively occupied by specific dominant species. All five dominant species recorded RAI values exceeding 1.36, significantly surpassing other co-occurring avian species (Suppl. material 2).

**Figure 3.**
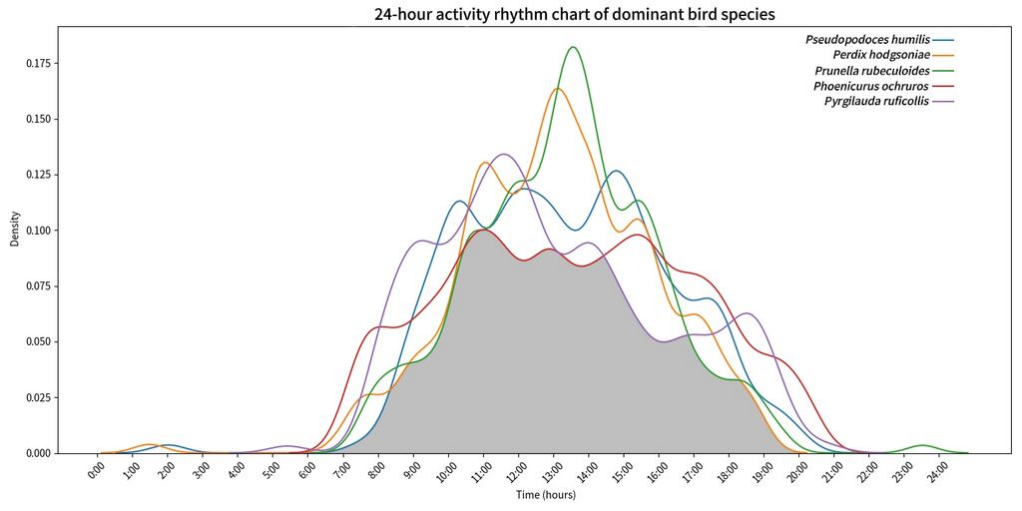
A comparison of diurnal and nocturnal activity patterns of five bird species over a 24-hour cycle, where the shaded areas represent the overlap of the activity pattern curves, indicating that these five bird species have overlapping active times during this period.

The diel activity rhythms of the dominant bird species, Ground Tit (*Pseudopodoceshumilis*), Tibetan Partridge (*Perdixhodgsoniae*), Robin Accentor (*Prunellarubeculoides*), Black Redstart (*Phoenicurusochruros*) and Rufous-necked Snowfinch (*Montifringillaruficollis*), all exhibited a predominantly unimodal pattern. The activity peak for the Ground Tit occurred between 10:00 and 15:00 h, for the Tibetan Partridge between 12:00 and 14:00 h, for the Robin Accentor between 13:00 and 14:00 h, for the Black Redstart between 11:00 and 16:00 h and for the Rufous-necked Snowfinch between 11:00 and 12:00 h (Fig. 3).

**Figure 4.**
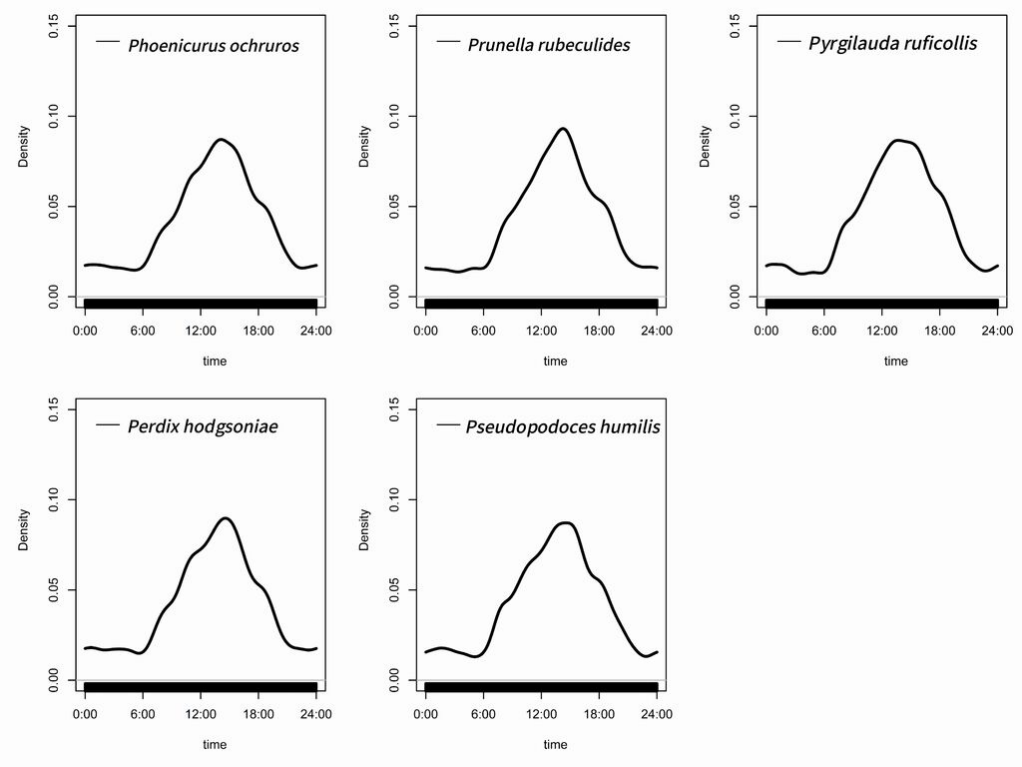
The activity time distribution of five dominant species in Baqing County is represented by kernel density curves, reflecting the relative frequency of bird species activity at a specific point in time.

Kernel density estimation was used to calculate overlap coefficients (Δ) of activity rhythms amongst species, revealing significant variation in temporal niche overlap amongst species pairs. The highest activity overlap coefficient observed was 0.9270 between *Perdixhodgsoniae* and *Prunellarubeculoides*, indicating similar diel activity patterns. Significant activity overlap was also observed between *Pseudopodoceshumilis* and *Perdixhodgsoniae* (Δ = 0.8758), *Pseudopodoceshumilis* and *Prunellarubeculoides* (Δ = 0.8713), *Pseudopodoceshumilis* and *Phoenicurusochruros* (Δ = 0.8627) and between *Perdixhodgsoniae* and *Pyrgilaudaruficollis* (Δ = 0.8085). Relatively lower overlap coefficients were recorded between *Phoenicurusochruros* and *Prunellarubeculoides* (Δ = 0.7995) and between *Prunellarubeculoides* and *Pyrgilaudaruficollis* (Δ = 0.7813). Additionally, the overlap coefficient between *Phoenicurusochruros* and *Pyrgilaudaruficollis* was 0.8579, representing a moderately high level (Fig. 4).

### Spatial distribution analysis

**Figure 5.**
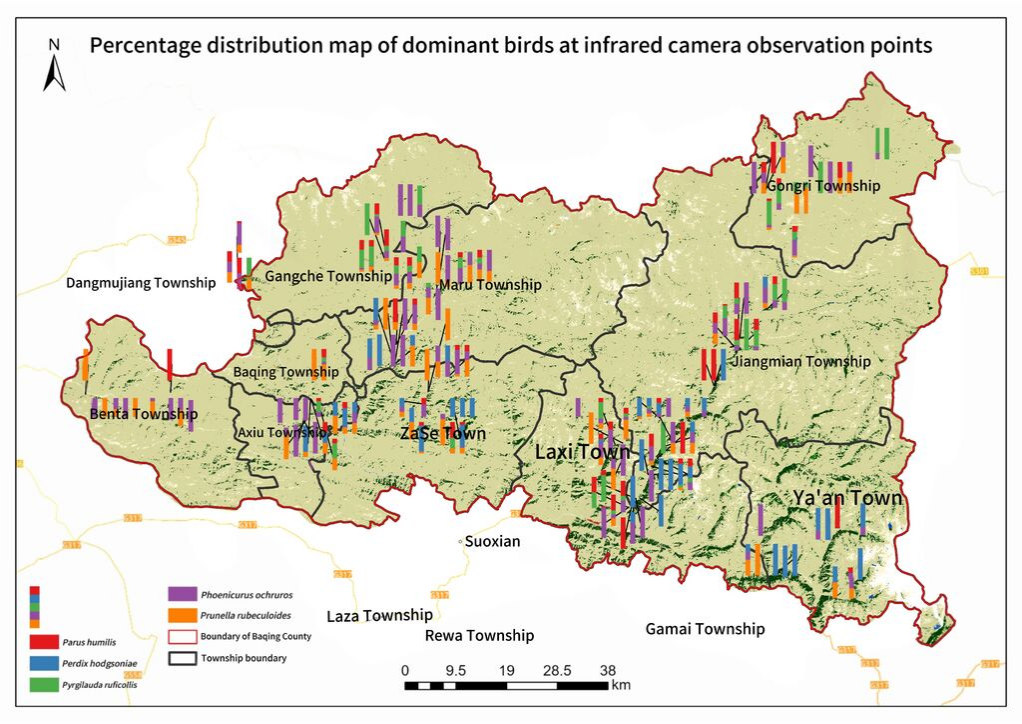
Proportion distribution of dominant bird species at each camera trap, with different coloured bars indicating the proportion of valid photographs of dominant bird species monitored by infrared cameras to the total number of photos at the site.

Amongst the bird species recorded by infrared cameras, the five most frequently documented were Black Redstart (*Phoenicurusochruros*), Robin Accentor (*Prunellarubeculoides*), Rufous-necked Snowfinch (*Pyrgilaudaruficollis*), Tibetan Partridge (*Perdixhodgsoniae*) and Ground Tit (*Pseudopodoceshumilis*). Black Redstart was recorded at 98 camera sites, representing 43.95% of all monitoring locations and constituting approximately half of all valid wildlife captures. This species exhibited significantly higher relative abundance compared to the other four species: Robin Accentor (33.18%), Rufous-necked Snowfinch (20.18%), Tibetan Partridge (19.28%) and Ground Tit (26.91%). Each of these four species was documented at over 40 camera sites. The findings indicate that the Black Redstart exhibited the most extensive distribution range, followed by the Robin Accentor. Due to the strong flight capabilities of birds, their distribution areas showed significant overlap, with only limited regions exclusive to certain dominant bird species (Fig. 5).

**Figure 6.**
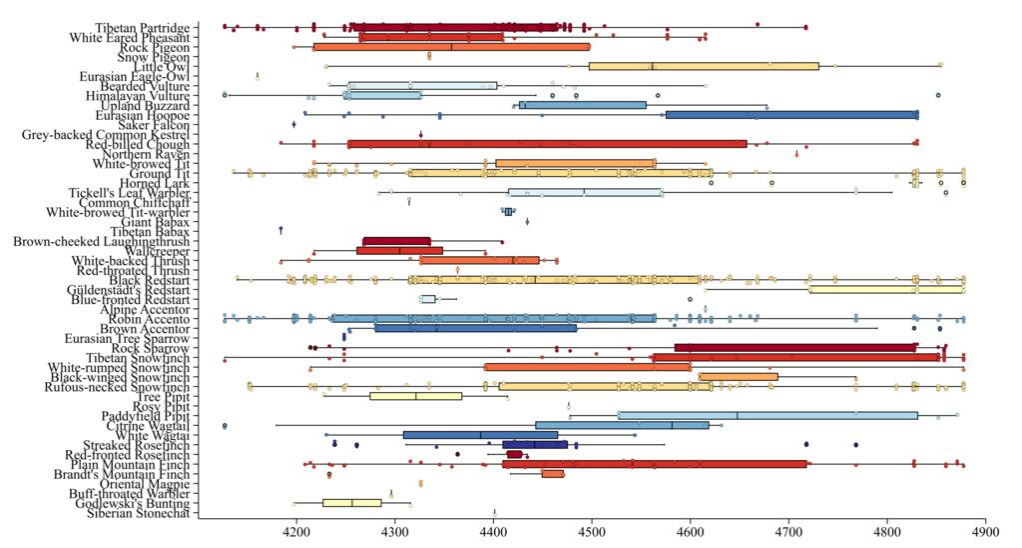
Altitudinal distribution of bird species occurrences recorded by camera trapping. Boxes represent interquartile ranges, whiskers extend to 1.5 times the interquartile range and points show individual records.

Analysis of altitudinal distribution revealed that five species were photographed only above 4,600 m, 30 species were found only below 4,600 m and 16 species were distributed in both altitude ranges. The Brown Accentor (*Prunellafulvescens*), Tibetan Partridge (*Perdixhodgsoniae*), Tickell's Leaf Warbler (*Phylloscopusaffinis*), Streaked Rosefinch (*Carpodacusrubicilloides*) and Citrine Wagtail (*Motacillacitreola*) exhibit a larger altitudinal range difference, ranging from 500 to 750 m. Amongst these species, Northern Raven (*Corvuscorax*), Horned Lark (*Eremophilaalpestris*), Güldenstädt's Redstart (*Phoenicuruserythrogastrus*), Alpine Accentor (*Prunellacollaris*) and Black-winged Snowfinch (*Montifringillaadamsi*) are distributed at higher altitudes, above 4600 m. In contrast, the brown Brown-cheeked Laughingthrush (*Trochalopteronhenrici*), Wallcreeper (*Tichodromamuraria*) and Godlewski's Bunting (*Emberizagodlewskii*) inhabit lower altitudinal ranges, below 4400 m (Fig. 6).

**Figure 7.**
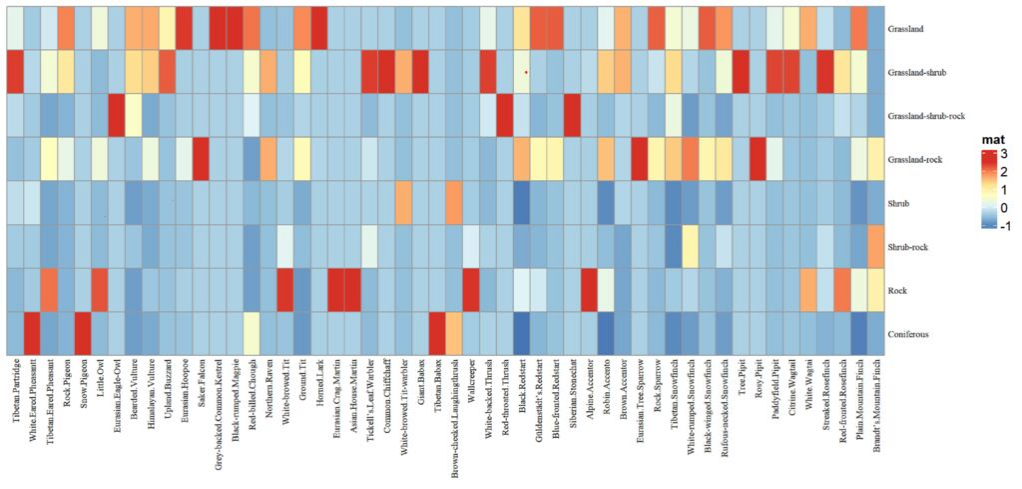
The heat map of habitat-effective photographs through captured species data.

Based on field survey conditions, the study area was classified into four primary habitat types (grassland, shrub, rock and coniferous forest) and four mixed habitat categories (grassland-shrub, grassland-shrub-rock, grassland-rock and shrub-rock). Analysis of five dominant bird species revealed their primary habitat preferences: Black Redstart (*Phoenicurusochruros*) predominantly inhabits grassland, rocky and grassland-shrub habitats; Robin Accentor (*Prunellarubeculoides*) occupies grassland and rocky habitats; Ground Tit (*Pseudopodoceshumilis*) primary habitat is in grassland habitats; Rufous-necked Snowfinch (*Pyrgilaudaruficollis*) frequents shrub-rock complexes; and Tibetan Partridge (*Perdixhodgsoniae*) utilises grassland and grassland-shrub-rock mosaics (Fig. 7, Suppl. material 3).

## Discussion

### Effects of survey methods on avian diversity

This study systematically monitored the bird community in Baqing County, Qinghai-Tibet Plateau, employing transect survey and camera trapping methods. The monitoring results revealed that, while both methods adequately documented common bird species within the survey area, neither achieved complete coverage of all avian species.

The transect survey demonstrated distinct advantages in recording waterfowl near aquatic habitats (e.g. Bar-headed Goose) and aerial foraging species (e.g. Golden Eagle). Conversely, camera trapping proved more effective in detecting cryptic species (e.g. Chinese Babax) and relatively rare birds (e.g. Red-throated Thrush). The primary advantage of the transect survey lies in its coverage of a larger survey area (Li et al. 2019); most birds inhabiting open terrains are readily observable. However, its limitations include shorter observation durations and potential disturbance to species behaviour, which may preclude accurate recording (Chen et al. 2016). Camera traps, typically 0.5 to 1.0 m above ground, capture birds only within their frontal detection zone (Zhang et al. 2018, Li et al. 2019). Their key strength is enabling long-term monitoring with minimal disturbance to species (Li et al. 2019). Therefore, integrating both survey methods compensates for their respective limitations, resulting in a more comprehensive inventory of avian species within the study area (Cheng 2023).

### Avian composition and biogeographical analysis

A total of 81 bird species were recorded in the study area, amongst which 64 species belong to the order Passeriformes, representing the highest species richness within the avian community. The birds of the order Passeriformes are the most diverse avian group on earth, accounting for 60% of all bird species and dominating in most habitats. Research shows that perching birds have better adaptation to high-altitude environments. This finding aligns with the results of avian surveys conducted in the TRSNP by Gao et al. (2019).

From a zoogeographical perspective, avian species in Baqing County are predominantly composed of the Highland type, Holarctic type, Palaearctic type and Himalayan-Hengduan Mountains type, with a minority classified as the Northeast-North China type, Northeast China type and Oriental type (Fan et al. 2020). The biogeographical distribution of birds reflects a natural pattern shaped by long-term evolutionary adaptation to geological history and contemporary ecological conditions (Song and Qu 2025). Since the Oligocene, the continuous uplift of the Qinghai-Tibet Plateau has led to increasingly arid and cold climatic conditions (Wang et al. 2018). Therefore, cold and drought-adapted groups (Liu et al. 2020), such as the Palaearctic and Highland types, have gradually become dominant in the plateau avian community (Fan et al. 2020, Girish and Srinivasan 2020), while warm and humidity-dependent groups (e.g. the Oriental type) experienced southward range contraction or local extinction due to their inability to adapt to the frigid environment (Freeman and Class Freeman 2014, Yang et al. 2021).

Under the current global warming trend, the harsh climate in high-altitude regions has begun to moderate. Studies indicate that the annual warming rate on the Qinghai-Tibet Plateau is approximately twice the global average (Cheng et al. 2019). Thus, Oriental and North China–Northeast China type species, such as Eastern Cattle Egret (*Bubulcuscoromandus*) and Brown Shrike (*Laniuscristatus*), may serve as pioneer species responding to climatic shifts. Moving forward, long-term species monitoring is essential to track the frequency of recurrent occurrences and the stability of newly-colonizing species within the study area over defined periods. Such efforts will clarify species' adaptive responses under changing climatic conditions (Mawdsley et al. 2009).

### Behavioural patterns of dominant birds across temporal scales

The activity rhythm of animals, a key dimension representing their temporal niche, exhibits significant resilience and high flexibility. This inherent temporal variation effectively mitigates competitive pressure and interference effects amongst multiple species co-existing within the same geographic area that share similar ecological niches (Li et al. 2021). Studies indicate that sympatric species can reduce competition through temporal segregation of activity peaks (Liu et al. 2017). However, due to the unique geographic characteristics of Baqing County, where the surveyed area experiences substantial diurnal temperature fluctuations (Jia et al. 2005), birds increase their activity frequency during periods of higher ambient temperatures to minimise energy expenditure. Consequently, the coefficient of overlap in daily activity patterns amongst dominant species is relatively high and niche differentiation in activity timing between species is not pronounced.

Analysis of daily activity rhythms in dominant species using infrared camera trap data revealed that, despite high temporal overlap during active periods, co-existence is facilitated through multi-dimensional niche differentiation (Feng et al. 2022). The Tibetan Partridge (*Perdixhodgsoniae)* and Robin Accentor (*Prunellarubeculoides*) exhibit the highest coefficient of overlap in daily activity rhythms. However, the Tibetan Partridge is a herbivorous bird primarily feeding on ground-level grass seeds (Que 2011), whereas the Robin Accentor is omnivorous, relying mainly on insects as its food source. Furthermore, the Tibetan Partridge prefers shrubland habitats (Dorge et al. 2014), while the Robin Accentor predominantly utilises rock crevices for nesting and breeding (Li et al. 2003). Niche differentiation in dietary composition, habitat strata and spatial utilisation buffers competition arising from overlapping activity times between these species (Shafaeipour et al. 2020).

Similarly, although the activity rhythm overlap coefficient between the Black Redstart (*Phoenicurusochruros*) and Rufous-necked Snowfinch (*Pyrgilaudaruficollis*) is relatively high (0.8579) and potential competition for food resources may exist, their vertical distribution ranges differ significantly. The former relies on natural cavities for breeding (Mu et al. 2008), while the latter utilises burrows of plateau pikas (*Ochotonacurzoniae*) (Zeng 2019). Thus, despite some overlap, competition is weaker and co-existence is further promoted by differences in habitat selection preferences.

### Habitat preferences and spatial distribution patterns of birds

Based on infrared camera monitoring data, the grassland-shrub and grassland-rock ecotones yielded the highest number of bird species recorded and also accounted for the highest number of valid bird photographs within grassland-associated habitats. The grassland area in the study region significantly exceeds that of other habitat types. According to the species-area effect, larger areas support a greater number of species (Rahbek 2005, Wang et al. 2023); consequently, the number of species utilising grassland-related habitats is higher than in other habitats. The edge effect indicates that ecotones, where two habitats intermingle, often feature complex environmental conditions and contain species from both parent habitats, as well as species unique to the ecotone itself. Therefore, the grassland-shrub and grassland-rock ecotones, as interfaces between two habitat types, exhibit markedly higher species richness than the grassland habitat alone (Foggo et al. 2001, Fahrig et al. 2011). Furthermore, shrubs and rocks provide birds with enhanced visibility for foraging and predator detection (Pithon et al. 2021), as well as concealment opportunities, contributing to the highest avian species diversity in these two ecotone habitats.

A combination of biotic and abiotic factors, including climate, vegetation habitat and evolutionary history, influences the vertical spatial distribution pattern of species diversity (García-Navas et al. 2021). In this study, the majority of bird species were primarily distributed at relatively low elevations below 4600 m, with only a few species found at altitudes above this level. Birds require stable temperature and moisture conditions to sustain their populations (Zhao et al. 2017). The topography of the study area slopes from higher elevations in the north to lower elevations in the south (Ma et al. 2023). The northern high-altitude zone experiences lower temperatures and its vegetation is dominated by sparse grasslands (Wang et al. 2018), which offer limited food resources for many bird species and which provide suboptimal conditions for nesting and reproduction. In contrast, the southern region has lower elevations and features diverse habitats such as coniferous forests, shrublands and wetlands, which create favourable living conditions for birds in the study area (Xu et al. 2009, Tan et al. 2020). However, human activities in this region are more frequent, potentially influencing the stability of the original ecosystem and causing significant damage to the existing biological community structure (Yang et al. 2021).

## Conclusions

The majority of bird species in Baqing County are found at elevations below 4,600 m, with only a few species occurring above this altitude. We recommend focusing on the distribution of birds within this elevation range and conducting further research on the factors influencing species distribution. Grassland and rock habitats, which cover extensive areas, exhibit high bird richness and are crucial for numerous bird species. Therefore, these habitats should be prioritised in avian conservation efforts. There is no significant difference in the temporal niche segregation of local resident birds in Baqing County regarding their daily activity patterns, which are linked to variations in habitat, diet and spatial use. Human activities, such as overgrazing, can lead to environmental pollution, food scarcity and impacts on bird reproduction. Therefore, it is essential to monitor and manage human activities in Baqing County and enhance public education on bird protection and biodiversity promotion. Our findings not only provide valuable insights for the conservation management of the TRSNP, but also offer a comprehensive scientific foundation for biodiversity protection on the Qinghai-Tibet Plateau.

## Supplementary Material

0CFAF572-B3D8-5FB5-B7E4-E3BB71A1F78A 10.3897/BDJ.13.e163095.suppl1 Supplementary material 1 Species list and their respective distribution types and residency types Data type Table File: oo_1373337.xlsx https://binary.pensoft.net/file/1373337 Jielei Xie

C012ABFC-8777-50EB-B53F-D164694B85DD 10.3897/BDJ.13.e163095.suppl2 Supplementary material 2 Relative abundance indexes based on infrared camera survey data Data type Table File: oo_1373354.xlsx https://binary.pensoft.net/file/1373354 Jielei Xie

BBBAEF0A-B659-56D6-AE31-CC6AAF93522C 10.3897/BDJ.13.e163095.suppl3 Supplementary material 3 Species habitat - Valid photo sheet Data type Table File: oo_1373356.xlsx https://binary.pensoft.net/file/1373356 Jielei Xie

CA4FF3BE-C338-52EB-BA48-7E528431E709 10.3897/BDJ.13.e163095.suppl4 Supplementary material 4 Species transect survey record form Data type Table File: oo_1373358.xlsx https://binary.pensoft.net/file/1373358 Jielei Xie

## Figures and Tables

**Figure 1.**
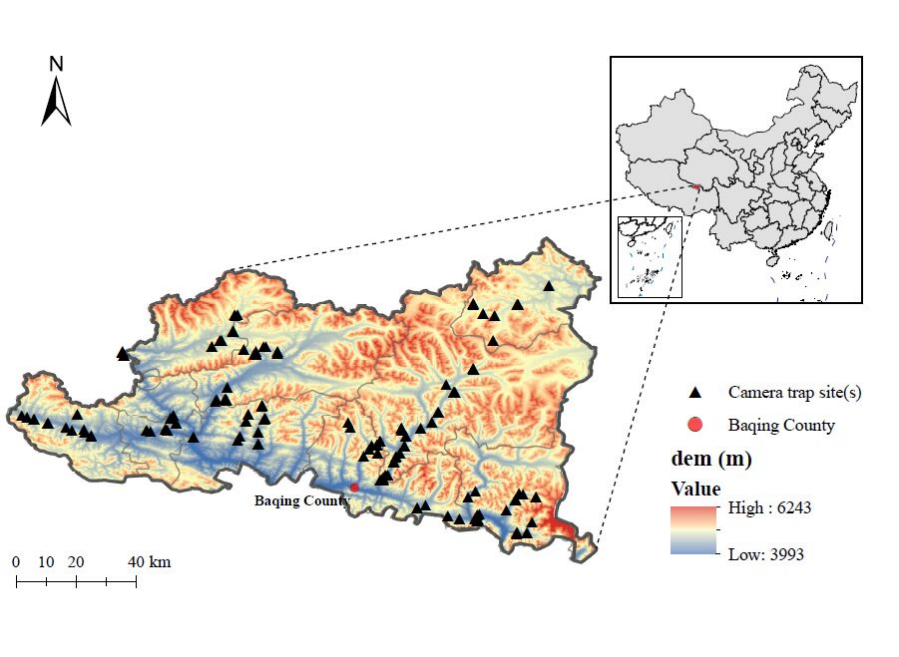
The layout diagram of camera trapping sites in Baqing County of Three-River-Source National Park.
